# Athletes’ Psychological Adaptation to Confinement Due to COVID-19: A Longitudinal Study

**DOI:** 10.3389/fpsyg.2020.613495

**Published:** 2021-01-14

**Authors:** Víctor J. Rubio, Iván Sánchez-Iglesias, Marta Bueno, Gema Martin

**Affiliations:** ^1^Department of Biological and Health Psychology, Universidad Autónoma de Madrid, Madrid, Spain; ^2^Department of Psychobiology and Behavioral Sciences Methods, Universidad Complutense de Madrid, Madrid, Spain; ^3^Sport and Exercise Psychology, Universidad Autónoma de Madrid, Madrid, Spain; ^4^Sport Psychology Unit, Center for Applied Psychology, Universidad Autónoma de Madrid, Madrid, Spain

**Keywords:** COVID-19, confinement, psychological adaptation, sport, physical activity, mental health

## Abstract

Studies of individuals under conditions of confinement or severe social and physical restrictions have consistently shown deleterious mental health effects but also high levels of adaptability when dealing with such conditions. Considering the role of physical activity and sport in psychological adaptation, this paper describes a longitudinal study to explore to what extent the imposed restrictions due to the outbreak of SARS-CoV-2 may have affected athletes’ mental health outcomes and how far the process of adaptation to confinement conditions is differentially affected depending on whether the sports activity was practiced individually or in a group, and outdoors, indoors, or both. Two hundred and seventy-four athletes were assessed over 7 weeks using the GHQ-28 and an *ad hoc* survey exploring the practice of physical activity. A mixed-model fixed effects ANCOVA was used to analyze the effects of time, place, and company in which the sport was practiced, with an index of the amount of physical activity expended as a covariate. Results show a significant effect of time in three out of four of the GHQ-28 subscales, in all cases showing a consistent adaptation to conditions over time. Results also show that playing sport indoors, outdoors, or both, and practicing alone vs. with others differentially affect the somatic symptoms exhibited during confinement: Athletes who practiced sport with others showed higher levels of somatic symptoms at the beginning of the set of data but a quicker rate of adaptation. Differences arising from practicing sport alone or with others were more pronounced in the case of indoor sports, which could be related to the fact that physical activity that can be practiced during confinement is more similar to that practiced indoors alone. Implications relating to what sport psychologists and other health professionals may offer to athletes in stressful situations are discussed.

## Introduction

Studies of individuals under conditions of confinement or severe social and physical restrictions have consistently shown deleterious mental health effects. This is the case of prisoners placed in solitary confinement. For instance, Chadick et al. (2018), using a pre-post design comparing a group of general population with people who had been up to 4 years in solitary confinement, found higher levels of depression, anxiety, post-traumatic stress, and somatic complaints in the latter. Valentine et al. (2019) reported a statistically significant association between length of segregation and mental illness outcomes; the longer the greater. Reiter et al. (2020), in a very recent study, found an extremely high rate of serious mental illness and self-harm of inmates in solitary confinement compared to the rest of the prison population. These results have also been confirmed in people facing other situations, which exposed them to medium or long periods of confinement and isolation, such as members of polar expeditions (Palinkas and Suedfeld, 2008) or astronauts on space missions (Suedfeld, 2005).

These isolated and confined scenarios are considered extreme and unusual environments due to their exotic, abnormal, and/or stressful nature, beyond the range of individuals’ optimal survival and/or implying conditions far removed from ordinary living conditions, generally involving high levels of stress (Suedfeld, 1987).

The outbreak of the Severe Acute Respiratory Syndrome Coronavirus Type 2 (SARS-CoV-2), responsible for COVID-19 at the end of 2019, and its rapid expansion worldwide leading to the declaration of pandemic by the World Health Organization, brought governments of many countries to order a lockdown, which included the banning of social gatherings, the closing of schools and colleges, restrictions on traveling, and the imposition of stay-at-home orders on people not directly involved in essential activities. All of these can be considered an extreme and unusual environment, according to Suedfeld (1987). In fact, the studies already carried out on the impact and disruption this situation is producing worldwide have highlighted its stressful nature. For instance, in one of the first studies carried out, Wang et al. (2020) reported an increase in depressive and anxiety symptoms and stress levels in a sample of Chinese people from different cities in China during the initial stages of confinement, and Zhang et al. (2020) found individuals reporting several mental and physical health issues; the larger the restrictions to their ordinary life, the worse their mental and physical health conditions, just one-month after the commencement of confinement in that country. The same occurred in other countries, as Ammar et al. (2020b) reported in an extensive study including 1,047 participants from a wide range of countries, mainly from Asia, Africa, and Europe. These authors found an expected decrease in social participation but also a substantial reduction in life satisfaction. Moreover, authors also detected an increase in the reporting of depressive symptoms and in the need for social support.

Human beings, as social animals, need sociality (Hawkley and Capitanio, 2015), and confinement, if not challenging to survival, was definitely producing a substantial change in communities’ ordinary way of living, seriously affecting their physical contact with other people and condemning many to a strict social isolation, not to mention the stress produced by uncertainty regarding one’s own and others’ health, jobs, and future way of living. In all cases, these situations are associated to an overlap between living and working environments, a non-desired separation from family and friends, a restriction on daily life activities including leisure activities, and forcing either physical closeness with cohabitants or full isolation for a period of time (Nicolas et al., 2019).

The crisis produced by the pandemic and confinement measures have also affected the practice of sport and physical activity (Ammar et al., 2020a; López-Bueno et al., 2020; Stanton et al., 2020), with competitions canceled and practicing physical activity out of doors banned or severely restricted at the height of the confinement. Precisely this reduction in physical activity, together with the cutback in social interactions, has been related to the increase in major sleep and psychological disorders (Chtourou et al., 2020; Lesser and Nienhuis, 2020; Stanton et al., 2020).

### Psychological Adaptation to Stressful Conditions

Despite the large amount of evidence regarding the adverse consequences of isolation and confinement, several authors have also called for a more salutogenic outlook when focusing on the psychological effects of facing such extreme and unusual environments (Suedfeld and Steel, 2000). One particularly important aspect is the adaptability human beings show when dealing with isolated, confined, and/or extreme conditions. Adaptability has been defined as “the capacity to make appropriate responses to changed or changing situations; the ability to modify or adjust one’s behavior in meeting different circumstances” (VandenBos, 2007). Likewise, many studies have used mental health as an outcome of adaptability [see Bartone et al. (2018), review on individual differences in adaptability to such conditions].

The idea of adaptation is not new in the field of psychology. According to Brickman and Campbell (1971), people usually tend to adapt to either strongly positive or strongly negative life events and are prone to return back to the baseline levels of subjective well-being. This idea has been tested through different pieces of research. For instance, Lucas et al. (2003), in a 15-year longitudinal study, surveyed over 24,000 16+-year-old German households assessing the effects of marital transitions on life satisfaction. Their findings showed that people tended toward stable levels of well-being and usually returned to their baseline level within a period of time after the event. However, they also appreciated individual differences among participants. As an example, those who reacted strongly to the events showed a slower return to baseline (Lucas et al., 2003).

### Relationships Between Physical Activity, Mental Health, and Psychological Adaptation

Physical activity has beneficial effects across a very broad range of physical health outcomes, including cardiovascular health, metabolic functioning, musculoskeletal balance, functional capacity, and general health (Haskell et al., 2007; García-Hermoso et al., 2020). It has also shown protective effects over diseases such as colon cancer, stroke, or diabetes (Shiroma and Lee, 2010; Loaiza-Betancur et al., 2020; Loh et al., 2020) and is directly related to preventing obesity and the insidious effects of sedentarism (Kim et al., 2020), the “silent enemy” (Bauman, 2004).

However, the positive effects of physical activity are not just limited to physical outcomes: acute and chronic exercise have been shown to increase several cognitive-related outcomes, such as executive functioning and academic performance (Chang et al., 2012; de Greeff et al., 2018). Furthermore, there is a well-documented relationship between physical activity and mood, both increasing positive affect and reducing negative affect (Chan et al., 2019). Regularly practicing physical activity is associated with higher levels of health-related quality of life (Penedo and Dahn, 2005) and treatments based on promoting exercise have also been shown to be effective in reducing stress (Schnohr et al., 2005), anxiety, and depression (Steptoe, 2006; Perry et al., 2020). These results have also been confirmed during the pandemic, the individuals reporting worse mood being either those who had reduced the frequency of their regular physical activity or those who had remained inactive if they did not exercise pre-pandemic (Brand et al., 2020). Moreover, there is also a relationship between exercise, well-being, and health (Grant et al., 2009). Additionally, there exists a closed-loop linking physical activity, well-being, and health (Steptoe, 2006, 2010).

Likewise, physical activity contributes to increasing adaptation to extreme and unusual conditions. For instance, Niebuhr et al. (2013), in a large study of more than 15,000 United States army recruits, found that physical conditioning predicted better adaptation of recruits during their first 6-month service period. In the same vein, Schneider et al. (2013) found endurance exercising to increase adaptability in a group of six participants confined in the MARS500 capsule. Authors also highlighted not just exercise *per se* but the general immersive experience of practicing the activity (rhythmicity, refocusing, etc.) as the key factor contributing to adaptability.

The crisis produced by the pandemic and confinement has also affected the practice of sport and physical activity (Ammar et al., 2020a; López-Bueno et al., 2020; Stanton et al., 2020), with competitions canceled and practicing physical activity out of doors banned or severely restricted at the height of the confinement. Precisely this reduction in physical activity together with the cutback in social interactions have been related to the increase in major sleep and psychological disorders (Chtourou et al., 2020; Lesser and Nienhuis, 2020; Stanton et al., 2020). Additional stressors to athletes are career disruption, uncertainty regarding major competitions and qualifying tournaments, and limitations to accessing training facilities (di Fronso et al., 2020; Schinke et al., 2020).

Physical activity in children, adolescents, and young adults is achieved mainly through practicing sports and is related to energy liberation, friendship, and enjoyment. Therefore, the restrictions imposed due to the lockdown to check the spread of COVID-19 have severely affected the exercise to which athletes and sportspeople were accustomed. Nevertheless, confinement conditions do not equally affect all kinds of sports and/or physical activity. The imposed lockdown might have had a more severe impact on sports and physical activity carried out outdoors and with other people (e.g., soccer) and less severe on indoor activities that are usually practiced alone (e.g., calisthenics).

This study longitudinally explores the process of psychological adaptation to the confinement conditions. As mentioned, the psychological adaptation process can be expressed in terms of the extent to which the restrictions may affect athletes’ mental health outcomes. The study is also focused on ascertaining how far these mental health outcomes are differentially affected according to the type of sport (individual or group) and the place (outdoors, indoors, or both) where it was practiced. As Brickman and Campbell (1971) suggested, it is expected that athletes will show a progressive reduction in mental health disorder outcomes over time, thus characterizing a process of psychological adaptation to the conditions imposed by the pandemic. However, even though the study is exploratory in nature, the adaptation process to confinement is also expected to be affected differently depending on the impact of restrictive measures on the sport activity: greater restrictions to the ordinary performance of the sport activity give rise to more limited contact with mates, worse mental health outcomes, and slower psychological adaptation to the situation.

## Materials and Methods

### Participants

The sample comprises 274 participants (52.2% female) with ages between 18 and 73 years (*M* = 35.8, *SD* = 14.1). Inclusion criteria to participate in the study were as follows: (1) being 18+ years old, (2) being residents of Spain, and (3) practicing physical activity or sport a minimum of 1 h a day at least 3 days a week. Using a snowball sampling approach, participants were recruited through social networks (WhatsApp, Instagram, Twitter, and Facebook) where they were invited to participate in the study and to disseminate the link to acquaintances who may be interested in participating as well.

### Instruments

#### Spanish Version of the Goldberg and Hillier (1979) General Health Questionnaire-28 (GHQ-28) (Lobo et al., 1986)

This is a symptom screening instrument for differentiating psychiatric from non-psychiatric patients, also used in epidemiological studies to identify somatic, anxiety, social dysfunction, and depression symptoms, which make up the seven-item each, four-factor questionnaire. Participants are asked about “how their health has been in general over the past week” and items are answered on a four-level Likert-type scale from “Better than usual” to “Much worse than usual.” According to the Spanish adaptation, which uses what has been called the classic scoring method (see Campbell et al., 2003), they are scored as 0 or 1 based on the symptom being compatible or not with psychopathology. The scores for each factor ranged from 0 to 7; the higher the score, the more psychopathological symptoms shown. Examples of items are as follows: Have you recently… Been feeling perfectly well and in good health? (Somatic symptoms), …Lost much sleep over worry? (Anxiety and insomnia), …Been managing to keep yourself busy and occupied? (Social dysfunction), …Been thinking of yourself as a worthless person? (Severe depression).

The psychometric characteristics of the scale have been demonstrated adequate in the original adaptation and in other studies (Lobo et al., 1986; Godoy-Izquierdo et al., 2002). We assessed reliability in our sample *via* Cronbach’s alpha, at each week of measurement. Depression showed low consistency at some measurement moments, with values ranging from 0.366 to 0.714. However, we found adequate reliability values for the other three subscales, ranging from 0.691 to 0.802 for Somatic Symptoms, 0.810 to 0.847 for Anxiety, and 0.779 to 0.835 for Social Dysfunction.

### Sports Activity and Sociodemographic Survey

In the first wave, an *ad hoc* survey was designed to collect information about participants’ age and gender as well as how many days a week they practiced sport, for how long each day (1 h, between 1 and 2 h, between 2 and 3 h, and more than 3 h), perceived intensity (light, moderate, and strong), whether the activity was individual or with others (individual sport, team sport, physical activity practiced alone, physical activity practiced with others, and more than one option), and what kind of activity. The following waves collected information about physical activity practiced during confinement (yes or no, how many days, for how long each day, degree of intensity, and kind of activity) and about the conditions and changes in confinement in the participants’ area of residence.

### Index of Quantity of Sports Practice

An Index of Quality of Sports Practice (ioQ) was computed based on subjects’ reports on how many days a week they usually practiced sports or physical exercise before confinement, and the duration of each session. The IoQ was obtained by combining (i.e., multiplying) these two variables, with scores potentially ranging from 1 to 28. In our sample, this index ranged from 3 to 24 (*M* = 8.7, *SD* = 4.8).

### Procedure

The study was carried out over 7 weeks extending from the beginning of April to May 2020, concurring with the establishment of tighter measures by the Spanish government on the whole country, their easing, and subsequent return to a “new normality.”^1^ Participants were asked to fill in the questionnaires online once a week on the Qualtrics platform. Athletes recruited using social networks who completed the first wave, including their e-mail address, received an e-mail with a link to the next series each Sunday morning at 8:00 am and were invited to access Qualtrics and answer the questions up until Monday at 11:59 pm. Participants who did not answer during Sunday received a reminder on Monday.

In order to protect the anonymity of participants’ responses, they were asked on accessing the survey for the first time to create a code including their initials and the last numbers of their ID, which was used to associate their responses to the survey in the different waves.

### Data Analysis

We calculated the descriptive statistics mean for the sample characteristics (gender, age, weekly frequency, intensity, duration, place, and company of exercise; see Participants). Polynomial *F* contrasts were carried out to test the linear relationship between weekly frequency and intensity or duration of the exercise.

For each factor in the GHQ (Somatic Symptoms, Anxiety, Social Dysfunction, and Depression), we used a mixed-model fixed effects ANCOVA to analyze the effects of time (7 weeks), place (indoors, outdoors, or both), and company (alone, with others), and their potential interactions, on GHQ scores, using the IoQ of sport practice, age, and gender as covariates (control variables). The Bonferroni correction was applied to pairwise *post hoc* tests. The mixed random effects procedure can include cases with incomplete data, effectively handling missing values (Pardo and Ruiz, 2012, p. 92; Quené and van den Bergh, 2004). We selected compound symmetry as the covariance matrix structure, assuming that (1) all the time point measures have the same variance, and (2) there is symmetry (i.e., the same covariance between each pair of time point measures). Significance level was set at α = .050. All statistical analyses were performed using SPSS 25.

## Results

The participants reported taking exercise between 3 and 7 days per week (*M* = 4.4, *SD* = 1.1), mildly (2.9% of participants), moderately (56.9%), or vigorously (40.1%); a higher frequency per week was associated with vigorous exercise, *F*(1, 271) = 47.92, *p* < 0.001, and η^2^ = 0.147. As for duration, participants exercised from 30 min to 1 h (29.9%), from 1 to 2 h (54.0%), from 2 to 3 h (12.8%), and more than 3 h (3.3%); a higher frequency per week was associated with higher duration, *F*(2, 270) = 41.87, *p* < 0.001, and η^2^ = 0.133. Thus, participants who exercised more often tended to do so more vigorously and in longer sessions.

We classified the sports activities reported by the subjects regarding (a) where they performed them: indoors (137 participants, 50.0%), outdoors (70 participants, 25.5%), or both indoors and outdoors (67 participants, 24.5%); and (b) the company: alone (154 participants, 56.2%) or with others (120 participants, 43.8%). Table 1 summarizes the activities reported.

**TABLE 1:** Sportive activities reported by the participants.

**Sportive activity**	**Responses**	**% of responses**	**% of cases**
Gym, fitness, dumbbells, TRX, crossfit, HIIT, GAP, and calisthenics	77	19.00%	28.10%
Jogging and trail running	46	11.30%	16.80%
Soccer, football, and futsal	40	9.90%	14.60%
Yoga, pilates, body balance, and bowspring	37	9.10%	13.50%
Swimming	35	8.60%	12.80%
Aerobic, zumba, body pump, and body combat	27	6.70%	9.90%
Biking	20	4.90%	7.30%
Tennis, paddle, and badminton	19	4.70%	6.90%
Trekking, walking, and hiking	17	4.20%	6.20%
Basketball	13	3.20%	4.70%
Athletics and triathlon	12	3.00%	4.40%
Dancing	11	2.70%	4.00%
Martial arts: karate, judo, taekwondo, boxing, and wrestling	10	2.50%	3.60%
Rugby	7	1.70%	2.60%
Lifeguard training	6	1.50%	2.20%
Water polo	5	1.20%	1.80%
Indoor cycling	4	1.00%	1.50%
Weightlifting, powerlifting, and power building	4	1.00%	1.50%
Climbing	2	0.50%	0.70%
Rehabilitation	2	0.50%	0.70%
Rowing, canoeing, and kayaking	2	0.50%	0.70%
Equestrianism	1	0.20%	0.40%
Fencing	1	0.20%	0.40%
Gymnastics	1	0.20%	0.40%
Ping-pong	1	0.20%	0.40%
Skating or skateboarding	1	0.20%	0.40%
Skiing	1	0.20%	0.40%
Surfing	1	0.20%	0.40%
Training for physical fitness test	1	0.20%	0.40%
Volleyball	1	0.20%	0.40%
Not specified	1	0.20%	0.40%
Total	406	100.00%	148.20%

**N* = 274 The total number of responses is greater than *N*, as each participant could report more than one activity.*

### Somatic Symptoms

Table 2 shows the estimated means and standard errors for the GHQ Somatic Symptoms values along measurements.

**TABLE 2:** GHQ somatic symptoms.

		**Week**						
		**1**	**2**	**3**	**4**	**5**	**6**	**7**
	
**Place**	**Company**	***M* (*SE*)**	***M* (*SE*)**	***M* (*SE*)**	***M* (*SE*)**	***M* (*SE*)**	***M* (*SE*)**	***M* (*SE*)**
Indoor	Alone	0.78 (0.09)	0.57 (0.09)	0.50 (0.09)	0.39 (0.10)	0.37 (0.10)	0.44 (0.10)	0.41 (0.10)
	With others	1.08 (0.11)	0.79 (0.12)	0.97 (0.12)	0.64 (0.13)	0.54 (0.14)	0.43 (0.13)	0.61 (0.14)
Outdoor	Alone	0.69 (0.13)	0.39 (0.14)	0.50 (0.14)	0.39 (0.15)	0.32 (0.15)	0.19 (0.15)	0.54 (0.15)
	With others	1.05 (0.14)	0.79 (0.15)	0.63 (0.15)	0.59 (0.16)	0.39 (0.16)	0.28 (0.17)	0.11 (0.17)
Both	Alone	0.69 (0.14)	0.52 (0.15)	0.35 (0.15)	0.30 (0.15)	0.32 (0.15)	0.33 (0.16)	0.32 (0.16)
	With others	0.66 (0.14)	0.41 (0.15)	0.38 (0.15)	0.22 (0.15)	0.25 (0.15)	0.34 (0.16)	0.68 (0.16)

*Estimated Means and Standard Errors Along Time, by Company and Place of Sportive Practice.*

The mixed model yielded the following results: As per the controlled variables, IoQ had a significant effect, *F*(1, 240.8) = 6.36, *p* = 0.012; age did not, *F*(1, 235.3) = 2.17, *p* = 0.142; and gender was also significant, *F*(1, 242.9) = 10.84, *p* = 0.001.

Regarding fixed effects, whereas place and company did not show a significant effect on Somatic Symptoms in the GHQ, *F*_*Place*_(2, 246.1) = 1.81, *p* = 0.165; *F*_*Company*_(1, 243.4) = 1.72, *p* = 0.191; time did, *F*_*Week*_(6, 1187.7) = 16.51, *p* < 0.001.

When the participants practiced both indoor and outdoor sports activities, there were no significant mean differences along time in any level of company, nor between company levels in any given week (all *p*s > 0.050).

**FIGURE 1:**
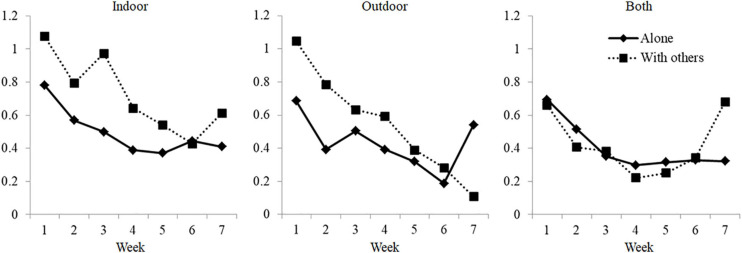
Estimated means of the GHQ somatic symptoms scores along time, by place and company of sports practice.

The two-way interactions were non-significant, *F*_*Place* × Company_(2, 244.8) = 0.54, *p* = 0.582; *F*_*Place* × Week_(12, 1,188.1) = 1.06, *p* = 0.396; *F*_*Company* × Week_(6, 1,188.3) = 0.80, *p* = 0.567. However, we found a three-way interaction Place × Company × Week, *F*_*Place* × Company × Week_(12, 1,188.2) = 1.86, *p* = 0.035. Based on *post hoc* analyses, and as can be seen in Figure 1, this three-way interaction shows that individuals were progressively adapting to the conditions through time, the later the better. However, adaptation was not the same depending on where (indoors, outdoors, or both) and with whom (with others or alone) the sport was practiced. Athletes who practiced sport with others, either indoors or outdoors (but not both indoors and outdoors), showed higher levels of somatic symptoms at the beginning of the set of data. Conversely, they showed a sharp negative slope, which is related to a quicker rate of adaptation. Differences arising from practicing sport alone or with others were more pronounced in the case of indoor sports, which could be related to the fact that physical activity that can be practiced during confinement is more similar to that practiced indoors alone (e.g., weightlifting at a gym) but more distant from sports played indoors with others (e.g., futsal).

### Anxiety

We found no effect of place and company on anxiety scores, *F*_*Place*_(2, 256.4) = 1.03, *p* = 0.358; *F*_*Company*_(1, 253.6) = 0.86, *p* = 0.353, but we found a main effect of time, *F*_*Week*_(6, 1,189.4) = 19.27, *p* < 0.001, and *post hoc* analyses showed a significant reduction in anxiety symptoms through time. We did not find interaction effects, either, *F*_*Place* × Company_(2, 255.3) = 0.41, *p* = 0.960; *F*_*Place* × Week_(12, 1,189.9) = 0.65, *p* = 0.792; *F*_*Company* × Week_(6, 1,190.0) = 0.73, *p* = 0.626; *F*_*Place* × Company × Week_(12, 1,189.9) = 0.57, *p* = 0.871, from which we infer that this reduction appears regardless of place and company in which sport is conducted. The covariate IoQ had a significant effect on anxiety, *F*(1, 249.8) = 6.60, *p* = 0.011, as well as on gender, *F*(1, 253.1) = 11.06, *p* = 0.001, but not on age, *F*(1, 246.1) = 1.35, *p* = 0.247. Table 3 shows the estimated descriptive statistics, by place, company, and time.

**TABLE 3:** GHQ anxiety.

		**Week**						
		**1**	**2**	**3**	**4**	**5**	**6**	**7**
	
**Place**	**Company**	***M* (*SE*)**	***M* (*SE*)**	***M* (*SE*)**	***M* (*SE*)**	***M* (*SE*)**	***M* (*SE*)**	***M* (*SE*)**
Indoor	Alone	1.07 (0.11)	0.81 (0.11)	0.77 (0.11)	0.60 (0.12)	0.59 (0.12)	0.56 (0.12)	0.67 (0.12)
	With others	1.20 (0.14)	1.06 (0.15)	0.91 (0.15)	0.64 (0.16)	0.68 (0.16)	0.52 (0.16)	0.73 (0.17)
Outdoor	Alone	0.87 (0.16)	0.68 (0.17)	0.65 (0.17)	0.65 (0.18)	0.53 (0.18)	0.46 (0.18)	0.58 (0.18)
	With others	1.20 (0.17)	0.91 (0.18)	0.95 (0.18)	0.63 (0.19)	0.64 (0.20)	0.68 (0.20)	0.60 (0.20)
Both	Alone	1.09 (0.17)	0.63 (0.18)	0.64 (0.18)	0.57 (0.18)	0.57 (0.18)	0.33 (0.19)	0.19 (0.19)
	With others	0.99 (0.17)	0.81 (0.18)	0.74 (0.18)	0.67 (0.18)	0.34 (0.19)	0.27 (0.19)	0.48 (0.20)

*Estimated means and standard errors along time, by company and place of sportive practice.*

### Social Dysfunction

We did not find main effects on social dysfunction for place or company, *F*_*Place*_(2, 261.9) = 0.60, *p* = 0.550; *F*_*Company*_(1, 259.1) = 0.34, *p* = 0.560. However, there was a significant effect of time, *F*_*Week*_(6, 1190.2) = 18.31, *p* < 0.001; *post hoc* analyses show a decrease of mean social dysfunction scores along time, with a slight non-significant upturn at week 7. We did not find any interaction effects, *F*_*Place* × Company_(2, 260.9) = 0.17, *p* = 0.842; *F*_*Place* × Week_(12, 1,190.7) = 1.17, *p* = 0.299; *F*_*Company* × Week_(6, 1,190.7) = 1.34, *p* = 0.235; *F*_*Place* × Company × Week_(12, 1,190.7) = 0.88, *p* = 0.564. The covariate IoQ showed no significant effect on social dysfunction, *F*(1, 254.8) = 3.43, *p* = 0.065; nor did gender, *F*(1, 258.6) = 2.28, *p* = 0.133; however, age did, *F*(1, 252.1) = 7.62, *p* = 0.006. Table 4 shows the estimated descriptive statistics as a function of the factors.

**TABLE 4:** GHQ social dysfunction.

		**Week**						
		**1**	**2**	**3**	**4**	**5**	**6**	**7**
	
**Place**	**Company**	***M* (*SE*)**	***M* (*SE*)**	***M* (*SE*)**	***M* (*SE*)**	***M* (*SE*)**	***M* (*SE*)**	***M* (*SE*)**
Indoor	Alone	0.83 (0.11)	0.81 (0.11)	0.73 (0.11)	0.58 (0.11)	0.62 (0.11)	0.61 (0.12)	0.62 (0.12)
	With others	1.08 (0.13)	1.07 (0.14)	1.02 (0.14)	0.89 (0.15)	0.63 (0.16)	0.55 (0.15)	0.94 (0.16)
Outdoor	Alone	0.85 (0.15)	0.72 (0.16)	0.52 (0.17)	0.52 (0.17)	0.39 (0.17)	0.39 (0.17)	0.41 (0.18)
	With others	1.24 (0.16)	0.88 (0.18)	0.67 (0.18)	0.75 (0.19)	0.45 (0.19)	0.38 (0.19)	0.36 (0.19)
Both	Alone	1.01 (0.17)	0.82 (0.17)	0.62 (0.17)	0.68 (0.17)	0.71 (0.18)	0.37 (0.18)	0.52 (0.18)
	With others	1.04 (0.16)	1.02 (0.18)	0.76 (0.17)	0.61 (0.18)	0.40 (0.18)	0.59 (0.18)	0.64 (0.19)

*Estimated means and standard errors along time, by company and place of sportive practice.*

### Depression

We did not find any fixed effect or main effects on depression scores, *F*_*Place*_(2, 239.4) = 0.38, *p* = 0.685; *F*_*Company*_(1, 236.9) = 3.35, *p* = 0.069; *F*_*Week*_(6, 1,168.9) = 1.38, *p* = 0.221; or interaction effects, *F*_*Place* × Company_(2, 238.4) = 0.38, *p* = 0.684; *F*_*Place* × Week_(12, 1,169.4) = 0.71, *p* = 0.745; *F*_*Company* × Week_(6, 1,169.5) = 0.96, *p* = 0.883; *F*_*Place* × Company × Week_(12, 1,169.4) = 1.05, *p* = 0.404. The covariate IoQ had a significant effect on depression, *F*(1, 33.0) = 14.45, *p* < 0.001; unlike age, *F*(1, 229.9) = 0.46, *p* = 0.500; or gender, *F*(1, 236.3) = 3.03, *p* = 0.083. Table 5 shows the estimated descriptive statistics.

**TABLE 5:** GHQ depression.

		**Week**						
		**1**	**2**	**3**	**4**	**5**	**6**	**7**
	
**Place**	**Company**	***M* (*SE*)**	***M* (*SE*)**	***M* (*SE*)**	***M* (*SE*)**	***M* (*SE*)**	***M* (*SE*)**	***M* (*SE*)**
Indoor	Alone	0.09 (0.03)	0.08 (0.03)	0.11 (0.03)	0.08 (0.04)	0.08 (0.04)	0.07 (0.04)	0.10 (0.04)
	With others	0.09 (0.04)	0.16 (0.04)	0.11 (0.04)	0.08 (0.05)	0.12 (0.05)	0.09 (0.05)	0.09 (0.05)
Outdoor	Alone	0.04 (0.05)	0.05 (0.05)	0.02 (0.05)	0.03 (0.05)	0.02 (0.05)	0.02 (0.05)	0.02 (0.06)
	With others	0.18 (0.05)	0.14 (0.05)	0.11 (0.06)	0.07 (0.06)	0.08 (0.06)	0.05 (0.06)	0.05 (0.06)
Both	Alone	0.06 (0.05)	0.05 (0.05)	0.07 (0.05)	0.03 (0.05)	0.04 (0.06)	0.01 (0.06)	0.01 (0.06)
	With others	0.10 (0.05)	0.11 (0.06)	0.12 (0.05)	0.16 (0.06)	0.00 (0.06)	0.12 (0.06)	0.13 (0.06)

*Estimated means and standard errors along time, by company and place of sportive practice.*

### Discussion

The current study analyzes the psychological adaptation process shown by athletes and people accustomed to the regular practice of physical activity during COVID-19 confinement in terms of their mental health outcomes. According to our results, participants show higher levels of mental health distress at the beginning of the set, when confinement was imposed, which is in tune with the findings of some other studies (di Fronso et al., 2020), but also an adaptation through time, significantly reducing the scores over a period of 7 weeks. These results are in agreement with scholars who have praised human beings’ capacity for resilience (Masten, 2001) and highlight the tendency people usually show to adapt to either strongly positive or strongly negative life events and return to their baseline levels of subjective well-being (Brickman and Campbell, 1971; Clark and Georgellis, 2013).

Authors have also emphasized the wide range of individual differences shown by people in their adaptation to stressful situations (Lucas et al., 2003). Sport plays a key role in promoting mental health and well-being (Penedo and Dahn, 2005; Steptoe, 2006, 2010; Chan et al., 2019) and has been successfully used for reducing stress, anxiety, and depression (Schnohr et al., 2005; Steptoe, 2006; Perry et al., 2020). Therefore, the current study has also analyzed to what extent the characteristics of the sport/physical activity practiced (i.e., place and company) might influence individuals’ adaptation to confinement.

According to our results, engaging in sport indoors, outdoors, or both, and practicing alone vs. with others, differentially affects mental health outcomes shown during confinement, particularly in one of the subscales of the GHQ scores (somatic symptoms). People accustomed to practicing outdoor sports with others presented higher levels of somatic symptoms at the beginning of confinement compared to those used to practicing sport alone and indoors, though they also presented a sharper decrease in negative mental health outcomes. This can be related to the fact that confinement restrictions are at odds with practicing indoor sports with others, such as futsal, but not with practicing calisthenics, for instance. These results, however, do not fit with di Fronso et al.’s (2020) results, who did not find significant differences between individual and group sports. Nevertheless, our results show differences in the psychological adaptation shown by athletes that could be longitudinally analyzed. Moreover, di Fronso et al. studied perceived stress and psychobiological states, which are essentially related to emotional responses to a situational condition.

Likewise, it should be noted that all these results have been obtained by partialling out the effects of the amount of physical activity (IoQ) participants engaged in before lockdown, which are seen to have a significant effect in all the subscales. This means that the amount of physical activity is related to mental health outcomes, in tune with previous literature (Schnohr et al., 2005; Steptoe, 2006; Chan et al., 2019; Perry et al., 2020).

The current study is not without some strengths and limitations. Among its strengths, the study adopts a longitudinal approach to address the adaptability shown by athletes and sportsmen and women engaging in regular physical activity in an extreme and unusual environment such as the period of lockdown imposed due to the pandemic. Although adaptation is a dynamic process, most pieces of research on the topic have analyzed the process from a cross-sectional approach (Frederick and Loewenstein, 1999; Lucas et al., 2003). Similarly, studies on the factors potentially underlying the differences among individuals during this adaptation process have usually been centered on personality and other inner traits, regardless of the role played by other possible influences (Lucas et al., 2003). As for the role of exercise in well-being (Grant et al., 2009), this study focuses on the type of physical activity and sport and with whom it is practiced, making it more or less liable to being affected by lockdown conditions and, therefore, promoting more or less rapid adjustments to these conditions.

Among its limitations, we did not find any main or interaction effects on the Depression subscale. However, the GHQ Depression subscale shows a low internal consistency, which could mean that we are missing some interesting effect due to this low reliability.

Furthermore, the study does not assess any personality dimensions or other factors such as social support, which have been shown to influence the adaptation process (Cocking, 2017; Bartone et al., 2018). Moreover, mental health outcomes have been used as a measure of adaptation. Such outcomes are present in many studies observing people’s psychological adaptation. However, attending to the fact that the GHQ is usually applied to mental health screening, it may have failed to detect other subtle manifestations of lack of adaptation. GHQ is a widely used instrument due to its psychometric properties and because it is an easy-to-administer scale, which might not prevent participants, particularly in a follow-up study, from giving up due to boredom and demand. In any case, a number of participants abandoned the study and this may have biased the original sample.

It should also be mentioned that the categorization of sports as indoor/outdoor and individual/group has been based on the information reported by participants. Even though there are sports that are played either indoors or outdoors in natural surroundings, others can be practiced both as indoor and outdoor games (e.g., basketball). In the same vein, there are individual sports that are practiced in groups (e.g., cycling). Our classification is based on the information reported by participants who stated whether they usually practiced sport indoors/outdoors or on an individual/group basis.

Finally, it may have been of interest to keep the series ongoing. However, the return to the “new normality” and the reduction in the number of participants fulfilling the last waves suggested the cancellation of data collection.

The study has implications relating to what sport psychologist practitioners and other health professionals may offer to athletes in stressful situations such as this. For instance, it has shown that athletes, in general, tend toward a psychological adaptation to the stressful conditions they have to face, but there are important individual and group differences. This means that intervention programs can be designed to improve such psychological adaptation. Health professionals may focus not only on mitigating the deleterious effects of canceling or restricting sports activities, but also on dimensions such as time management, personal growth, health habits, and general coping strategies to life events (Andreato et al., 2020; Batey and Parry, 2020; di Fronso et al., 2020; Jukic et al., 2020). Moreover, promoting social links with coaches and peers that may explain the scarcity of other significantly different studies found on comparing individual vs. team sports (di Fronso et al., 2020) might also be a way of reducing psychological impact and promoting adaptation to stressful conditions such those imposed by the pandemic (Bertollo et al., 2020).

## Data Availability Statement

The raw data supporting the conclusions of this article will be made available by the authors, without undue reservation.

## Ethics Statement

The studies involving human participants were reviewed and approved by Subcomité de Ética. Facultad de Psicología. Universidad Autónoma de Madrid. The patients/participants provided their written informed consent to participate in this study.

## Author Contributions

VR designed the study, supervised the data analysis, and wrote the first draft. IS-I carried out the data analysis and contributed to the final manuscript. MB recruited athletes and proceeded with the different survey waves. GM contributed to the design of the study, supervised the data collection, and revised the final version. All authors contributed to the article and approved the submitted version.

## Conflict of Interest

The authors declare that the research was conducted in the absence of any commercial or financial relationships that could be construed as a potential conflict of interest.

## Footnotes

1 Confinement in Spain started on 15 March. On 30 March, when the study began collecting data, confinement measures were tightened, and remained till 11 May, when de-escalation commenced and ended on 21 June.
